# Medico-Chirurgical Society of Edinburgh

**Published:** 1846-07

**Authors:** 


### Medico-Chirurgical Society of Edinburgh. Nov. 12, 1845.

*Contagiousness of Puerperal Fever.*—Dr. Peddie read a series of cases, illustrative of the contagiousness of puerperal fever, and its intimate connection or association with erysipelatous and phlebitic inflammation.

He had felt it to be his duty to communicate the facts connected

M. Flourens evidently perceives that these phenomena are opposed to the view which he maintains in the first part of his work, and to which we have previously referred. And he endeavours to account for these movements on the principle of reproduction of parts, and the relation which the division of the chord bears to the origin of the eighth pair of nerves. But none of these principles can ever account for that *positive power* which the animal possesses, of not merely moving its limbs, but, as we have shown, of taking cognisance of a stimulus, after division of the chord below the second pair of spinal nerves.

with these cases to the profession, as, besides being important in a pathological respect, they might perhaps contribute in some degree to avert hazard from a most interesting class of patients, and preserve to the medical man that peace of mind and prosperity in practice which might otherwise be interrupted. While candor required this course, he felt assured of obtaining sympathy on account of the painful situation in which he had been placed ; and that, though the unfortunate medium of spreading a fatal disease, no larger share of blame would be imputed to him than appeared due, when the history of these melancholy cases had been carefully considered.

Case i.—Mrs. S., aged 32, previously weak in health, and depressed in spirits, entertaining a presentiment of approaching death, was delivered on the 2d of September, after an easy labour. She was not carefully nursed; was seized on the third day with fever, which soon assumed the principal features of the malignant adynamic type, as described by Locock and others ; and died on the 10th, the eighth day from her accouchement.

Case ii.—Mrs. W., aged 23, a poor woman, attended for another practitioner, at that time out of town. She was a very delicate person, and predisposed also to fever by a strong presentiment of death; was delivered after a very easy labour, although her first child, on the 7th of September, and afterwards transferred from Dr. Peddie's care. She had begun to complain on the third day, and died on the 13th,—the sixth day from her accouchement,—with all the symptoms of the adynamic fever, complicated with much intestinal irritation. Dr. Peddie was not aware of this person's illness and death until after he had delivered his next patient.

Case iii.—Mrs. K., aged 25, also delicate, and in extremely low spirits, was delivered on the 14th September, of her second child ; labour natural and easy ; fevered on the 16th. Her case afterwards presented nearly the same features as those in Case 1, and she died on the 21st, seven days from the period of her confinement.

On the appearance of fever in Mrs. K.'s case, and finding that no epidemic prevailed, Dr. Peddie perceived that a contagious puerperal fever had broken out in his practice, and immediately consulted with several medical friends as to whether he should now for a timegive up all obstetric engagements. The advice received was to comply as usual with the next call for attendance, but to adopt every possible precaution against the transmission of the virus further. Accordingly, by assiduous attention to sprinkling and washing with the solution of the chloride of lime, proper arrangement of visits, and change of garments—not forgetting even the gloves and handkerchief —he secured the safe delivery and recovery of three patients, the first on the 19th, the next on the 22d, and the last on the 25th of September. That none of these patients showed the smallest tendency to fever, was the more satisfactory and encouraging, as the first and last were rather delicate, and the other was sister to Mrs. S., (Case No. 1,) who died only twelve days previously, and with whom she was much in contact. This, too, was the more singular, as she was in a state of so much alarm in the prospect of her own approaching hour of trial, as to be seized with labour rather prematurely. Dr. Peddie's anxious fears regarding the further 'propagation of the disease were thus lulled into security, and he felt disposed to view the occurrence of three consecutive cases of fever as one of those remarkable coincidences with which medical men occasionally meet; or if they really were instances of contagious fever, that the virulence of the morbific influence was exhausted, or could be overcome by the adoption of precautions. These sanguine hopes, however, were soon distressingly disappointed by the occurrence of the two following cases in rapid succession:—

Case iv.—Mrs. T., aged 29, was delivered of her second child at twelve at noon, on the 26th of September. Resided a few doors from Mrs. K., (Case No. 3,) whom she attended on the evening of her confinement, and visited frequently until the fever showed itself, and again on the 18th, although strictly prohibited, when she assisted in effecting a change of clothes and bedding. She was afterwards likewise exposed to contagion from the constant intercourse of friends between the two dwellings. Her labour was very easy, and she had every appearance of doing well, until next day at twelve o'clock, noon, when she fevered; and death occurred in the evening of the 30th, the third day from the period of accouchement.

Case v.—Mrs. T., aged 23, was delivered of her first child at twelve o'clock, noon, on the 27th of September, after a natural but rather tedious labour. It was completed some hours before Dr. Peddie had an opportunity of knowing that his last patient, (Case 4,) had been seized with the fever; and on making his evening visit, he found that she too was already affected with the dreadful malady. Death took place at three A. M., on the 30th, less than three days from the time of her confinement.

Dr. Peddie considered it beyond question, that Mrs. T., (Case 4,) had obtained contagious fomites from his last fatal case, (No. 3;) and while herself affected therefrom, had communicated the virus anew to his person, who conveyed it unconsciously to Mrs. T., (Case 5,) in whom it was developed almost from the moment of parturition.

Dr. Peddie now abandoned the practice of midwifery; was confined at home for several days, being much indisposed with sore throat, fatigue and anxiety; took medicine, and the warm bath; exposed the clothes worn at the cases in an airy chamber, and sprinkled them from time to time with the solution of the choloride of lime, and went into the country for eight days, four of which were spent at the seaside, and four on an excursion, into Perthshire and Stirlingshire. A

fortnight, less one day, thus elapsed before Dr. Peddie resumed his practice, and accepted (on the 13th Oct.,) the next obstetric call, in consequence of urgent solicitation. Dr. Peddie entered into a minute detail of the symptoms of this case, (Mrs. M.'s, aged 30, first child,) which unhappily proved fatal on the 24th of October, eleven days from the period of her accouchement; and he gave it as his own opinion, after much careful consideration, that he could not persuade himself of its having been a case of contagious puerperal fever, as there was a total dissimilarity in symptoms and mode of termination from the preceding characteristic cases, and as she had been in a most critical state of health for a considerable time previous to labour, with ulceration of the bowels, dilatation of the heart, and general debility. Dr. Peddie, however, stated, that lest his opinion was incorrect, he had felt it to be his duty to withdraw from midwifery practice for some time to come.

After some remarks on the nature of puerperal fever, and the opinion of authors concerning it, Dr. Peddie narrated several cases of erysipelas, phlebitis, and peritonitis, attended by him at the same time, and mixed up with his puerperal cases. From one of these, he thought it probable that the animal poison, producing the line of disastrous events in the accouchement chamber, originated, and referred, in proof of this opinion, to parallel instances related by Mr. Storr, of Doncaster, in the *Provincial Journal*; No. 166, 1843. The subject was a gentleman with a gangrenous erysipelas, spreading from sinuses surrounding the right hip-joint, which took their origin from a mismanaged bubo, and a much impaired constitution. It was the most malignant case of the kind ever witnessed by Dr. Peddie—proving fatal on the 13th of September, after the body had become deeply jaundiced, and large purulent deposits, with considerable emphysema, had formed in the right knee and left shoulder joints, as also among the muscles of the right forearm. This patient required dressings twice daily on account of the profuse discharge of dark-coloured fetid matter from the sinuses; and it was while attending him, although ablutions were regularly performed, that Dr. Peddie delivered Mrs. S., and Mrs. W., (Cases 1 and 2,) and, on the day following his death Mrs. K., (Case 3.)

Dr. Peddie then gave an account of several cases of *disease undoubtedly originating from the puerperal fever case*, No. 3,) thus affording a reflex proof of the existence of a puerperal contagious virus, affecting non-pregnant individuals, according to their special circumstances. One of them, a lady's nurse, who assisted frequently at Mrs. K's., was seized on the 25th of September with fever; the symptoms at first being chiefly referrible to the abdomen, and then to acute phlebitis of the right forearm, from which she had been bled, and died delirious on the 2d of October. Another was a nurse, who had acted occasionally at Mrs. K's., had also waited on the sick nurse for one day, and had visited Mrs. T., (Case 4,) on the afternoon of her confinement, was affected with erysipelas of the head and face, from which she recovered with difficulty. And a third was an old lady, who was lodging in the house of the lady's nurse, with whom she took fever simultaneously, which, however, in her case proved to be mild. It was also remarked, that almost every individual who had visited at Mrs. K.'s, during her illness, complained soon afterwards of one kind or another, particularly with slight feverishness and sore throat; and it was at this time also that Dr. Peddie himself became affected in the same way.

Dr. Peddie concluded his communication, by stating the following as the principal points which he thought the facts mentioned seemed to prove:—

1st. That a specific virus, of an animal nature, is produced under certain circumstances, and in turn generates a peculiar form of fever in the puerperal state.

2d. That a virus frequently originates from erysipelatous inflammation.

3d. When once generated, it may be communicated from one lying-in patient to another with extraordinary virulence, quite independently of locality or epidemic influence, either by direct intercourse, or through the medium of a third person; and that this is more likely to happen when the predispositions of a weak body and depressed mind exist.

4th. That it may also produce disease of various kinds in non-puerperal individuals, more especially of an erysipelatous and phlebitic character.

5th. That the treatment of a contagious puerperal fever, whether directed by theoretical opinions, or the indications of physical signs, proves of little avail; but that, if any theory is to be entertained respecting this malady, it should be that something of a specific and morbific nature requires to be thrown out of the system, and the powers of life at the same time sustained; and that the practice which holds out the greatest prospect, small at best, of this being accomplished, is the adoption of the diaphoretic and stimulant plans, according to the stage of the disease.

6th. That the principal concern of the medical man should be—seeing that a cure is so rare—to adopt every conceivable precaution against the occurrence of a single case of the disease, or to lesson the risk of its propagation when once established in his practice. And to attain these ends, patients in childbed should either not be attended at the same period with cases of malignant or severe erysipelas, or that proper caution should be observed as to ablutions, more especially after contact with any discharge from such patients; and when a case of puerperal fever does occur, chlorinated ablutions should be used; and if a second occur, he should withdraw from obstetric practice for two or three weeks, if possible; and in the interim attempt, by removal into the country, warm baths, and other alterative and purifying means, and by the exposure of clothing to a free atmosphere or high temperature, to rid himself of the subtle and powerful virus, which adheres to him so tenaciously.—*Northern Journal of Medicine.*

